# Essential Oil Composition of *Grindelia squarrosa* from Southern Idaho

**DOI:** 10.3390/molecules28093854

**Published:** 2023-05-02

**Authors:** Ambika Poudel, Noura S. Dosoky, Prabodh Satyal, Kathy Swor, William N. Setzer

**Affiliations:** 1 Aromatic Plant Research Center, 230 N 1200 E, Suite 100, Lehi, UT 84043, USA; apoudel@aromaticplant.org (A.P.); ndosoky@aromaticplant.org (N.S.D.); psatyal@aromaticplant.org (P.S.); 2 Independent Researcher, 1432 W. Heartland Dr., Kuna, ID 83634, USA; 3 Department of Chemistry, University of Alabama in Huntsville, Huntsville, AL 35899, USA

**Keywords:** curlycup gumweed, *serrulata*, gas chromatography, enantiomeric distribution, chiral

## Abstract

*Grindelia squarrosa* is an arid lands herb that has been used in Native American traditional medicine, is a potential source of pharmacologically active compounds, and has been explored as a source of biofuel. The purpose of this work was to examine the essential oil composition of *G. squarrosa* from southern Idaho. Gas chromatographic methods revealed the essential oil of *G. squarrosa* var. *serrulata* to be rich in monoterpenoids, α-pinene (21.9%), limonene (17.1%), terpinolene (10.6%), and borneol (6.5%). The essential oil composition of *G. squarrosa* from Idaho is similar to that previously reported from specimens collected from Montana and confirms the volatile phytochemistry of plants growing in North America. The major essential oil components were screened for antimicrobial activity against respiratory and dermal pathogens. (–)-β-Pinene showed strong antibacterial activity against *Streptococcus pneumoniae* (MIC 39.1 μg/mL) and (–)-borneol showed strong activity against *Staphylococcus aureus* (MIC 78.1 μg/mL).

## 1. Introduction

The genus *Grindelia* Willd. (Asteraceae) is made up of around 73 species [1], naturally ranging in western and central North America, Mexico, and South America, but introduced to eastern North America and the Old World [2]. *Grindelia squarrosa* (Pursh) Dunal (Asteraceae), curlycup gumweed, is a short-lived herb or subshrub, with oblong to oblanceolate, crenulate-serrulate leaves (1.5–7 cm long and 0.5–1.3 cm wide), and ranges throughout the Great Plains, Rocky Mountains, and Great Basin areas of North America. The plant has been introduced elsewhere [3,4], and has become an invasive species in central Europe [5]. Several varieties of *G. squarrosa* have been described [6], and three have been recorded in Idaho, namely *G. squarrosa* var. *quasiperennis* Lunell, *G. squarrosa* var. *serrulata* Steyerm., and *G. squarrosa* var. *squarrosa* Cronquist [7].

The Shoshoni Native Americans used *G. squarrosa* in their traditional medicine. A decoction of the plant was used as a cough medicine, as an antiseptic wash, and taken internally as an expectorant, to treat stomachache, smallpox, and measles [8]. Labdane diterpenoids (e.g., grindelic acid, 6-oxogrindelic acid, and 17-hydroxygrindelic acid) have been isolated and identified from *G. squarrosa* [9] and the plant has been investigated as a source of biofuel [10]. In this work, we hypothesize that the essential oil may contain components supporting the Native American use of the plant; we present the essential oil composition of the aerial parts of *G. squarrosa* var. *serrulata* collected from southern Idaho and antimicrobial screening of some major essential oil components.

## 2. Results and Discussion

**Figure 1:**
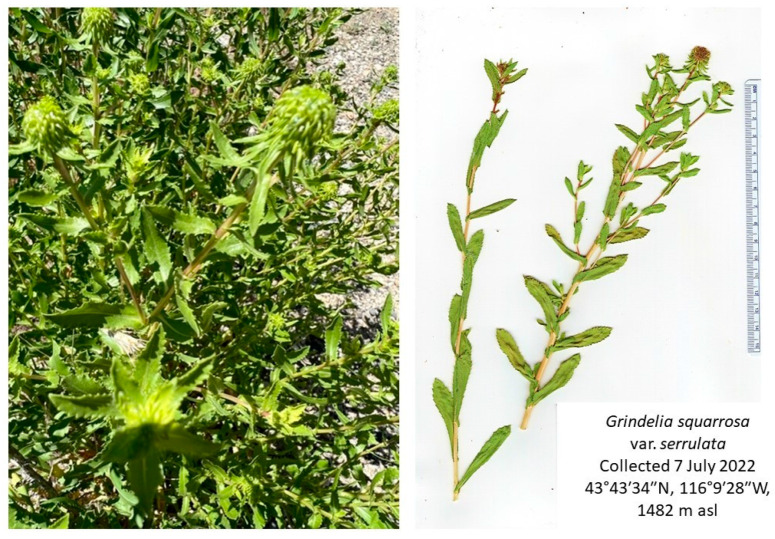
*Grindelia squarrosa* var. *serrulata* from southern Idaho. Photograph by K. Swor.

Based on botanical descriptions [11] and comparison with specimens from the New York Botanical Garden [7] the plant was identified as *G. squarrosa* var. *serrulata* (see Figure 1). Hydrodistillation of *G. squarrosa* aerial parts gave a colorless essential oil in 0.655% yield. Gas chromatographic analysis (GC-MS and GC-FID) revealed a total of 84 compounds (Table 1). The major components were α-pinene (21.9%), limonene (17.1%), terpinolene (10.6%), and borneol (6.5%).

There have been several reports on *Grindelia* essential oils, which are summarized in Table 2. Not surprisingly, there are obvious differences in essential oil compositions between *Grindelia* species. For example, *G. discoidea* was dominated by sesquiterpenoids [12], while *G. humilis* was rich in polyacetylenes [13], and *G. rubusta* had high concentrations of monoterpenoids [13,14,15]. There is variation in composition within species due to the geographical source of the plant material. For example, borneol dominated the essential oils of *G. rubusta* from Italy [14,15], but was apparently not detected in a sample grown in Germany [16]. Likewise, bornyl acetate was a major component in *G. squarrosa* from Romania (10.8%) [17], but was a relatively minor constituent in *G. squarrosa* from Germany (1.3% [13] and 0.7% [16]). It is not clear what factors may be involved in the differences in composition, but climatic, environmental, edaphic, seasonality, or genetic differences may be important.

Interestingly, the essential oil compositions of *G. squarrosa* from Idaho (this work) and from Montana [19] are similar in composition even though the locations are 424 km apart and on opposite sides of the Great Continental Divide of North America. Thus, for example, α-pinene (21.9% and 23.2% for the Idaho and Montana samples, respectively), limonene (17.1% and 14.7%), β-pinene (4.2% and 3.8%), and bornyl acetate (3.3% and 5.2%) concentrations are very similar. There are conspicuous differences in borneol (6.5% and 16.6%), terpinolene (10.6% and 2.0%), and *p*-cymen-8-ol (0.2% and 5.8%) concentrations between the two samples, however.

In order to determine the enantiomeric distributions of terpenoid components in *G. squarrosa* var. *serrulata*, the essential oil was subjected to chiral GC-MS (Table 3). The enantiomeric distributions for α-pinene, β-pinene, limonene, camphor, and borneol are comparable to those reported by Schepetkin and co-investigators, who found 100% (–)-α-pinene, 89% (–)-β-pinene, 98% (+)-limonene, 97% (–)-camphor, and 100% (–)-borneol in the sample from Montana [19]. As far as we are aware, there are no chiral GC analyses of essential oils of other *Grindelia* species.

The Native American traditional medicinal use of the plant as a cough medicine and as an antiseptic wash prompted investigation of the antimicrobial activities of the major essential oil components. The compounds α-pinene, β-pinene, limonene, borneol, and bornyl acetate were screened for antimicrobial activity against the respiratory and dermal pathogenic bacteria *Cutibacterium acnes*, *Staphylococcus aureus*, *Staphylococcus epidermidis*, *Streptococcus pneumoniae*, *Streptococcus pyogenes*, and the dermatophytic fungi *Microsporum canis*, *Microsporum gypseum*, *Serratia marcescens*, *Trichophyton mentagrophytes*, and *Trichophyton rubrum* (Table 4).

Based on previously published guidelines [20,21], essential oil components showing MIC values < 500 μg/mL should be considered as showing “strong activity”. Thus, *S. pneumoniae* was the most susceptible microorganism and *S. pyogenes* was the most resistant to the essential oil components. Notably, the major enantiomers, (–)-α-pinene, (–)-β-pinene, (+)-limonene, (–)-borneol, and (–)-bornyl acetate, generally showed broad antimicrobial activity. Furthermore, (±)-α-pinene and (–)-β-pinene have shown strong activity against methicillin-resistant *S. aureus* (MRSA) with IC_50_ values of 68.6 and 51.4 μg/mL, respectively [22], and both α-pinene and β-pinene (enantiomers not indicated) were active against *Klebsiella pneumoniae* with MIC values of 178 μg/mL and 170 μg/mL, respectively [23]. Terpinolene, not available for screening in this study, was found to be inactive (MIC >> 2000 μg/mL) against *C. acnes* and *S. aureus* [24]. Schepetkin and co-workers found that *G. squarrosa* essential oil as well as (−)-borneol activated human neutrophils [19]. Neutrophils play a critical role in inflammation. The antimicrobial activities of *G. squarrosa* essential oil components, coupled with the modulation of human neutrophil function of (−)-borneol, are consistent with Native American use of *G. squarrosa* to treat respiratory and dermal conditions.

## 3. Materials and Methods

### 3.1. Plant Material

Aerial parts of *Grindelia squarrosa* were collected from several plants growing wild near Bogus Basin Ski Resort, Idaho on July 7, 2022 (43°43′34″ N, 116°9′28″ W, 1482 m elevation). The plant was identified by W.N. Setzer. Based on botanical descriptions [11] and comparison with specimens from the New York Botanical Garden [7] the plant was identified as *G. squarrosa* var. *serrulata*. A voucher specimen (WNS-Gss-5718) has been deposited in the University of Alabama in Huntsville herbarium. The fresh plant material from several plants was combined and 91.11 g was hydrodistilled to give 597 mg of a colorless essential oil.

### 3.2. Gas Chromatographic Analysis

The essential oil of *G. squarrosa* var. *serrulata* was analyzed via gas chromatography mass spectrometry (GC-MS), gas chromatography with flame ionization detection (GC-FID), and chiral GC-MS as previously described in [25]. Briefly, gas chromatography—mass spectrometry (GC-MS) was carried out using a Shimadzu GC-MS-QP2010 Ultra (Shimadzu Scientific Instruments, Columbia, MD, USA). The mass selective detector was operated in the electron impact (EI) mode with an electron energy of 70 eV, a scan range of 40–400 atomic mass units and a scan rate of 3.0 scans per second, using the GC-MS solution software. The GC column used was a Zebron ZB-5ms fused silica capillary column (Phenomenex, Torrance, CA, USA), 60 m in length and 0.25 mm inner diameter; the stationary phase was (5% phenyl)-polydimethylsiloxane with a film thickness of 0.25 μm. The carrier gas was helium and the column head pressure was 208.3 kPa with a flow rate of 2.00 mL/min. The injector temperature was 260 °C, the interface temperature was 260 °C, and the ion source temperature was 260 °C. The GC oven temperature was programmed with an initial temperature of 50 °C; the temperature was increased to 260 °C at a rate of 2 °C/min, and then held at 260 °C for 5 min, for a total GC acquisition time of 110 min. The solvent cut time was set at 5 min. A 5% (*w*/*v*) solution of *G. squarrosa* var. *squarrosa* essential oil in dichloromethane was prepared and a volume of 0.1 μL was injected and the splitting mode was set at 24.5:1. Retention index (RI) values were determined by calibrating the instrument using a homologous series of *n*- alkanes using the logarithm-based arithmetic index method developed by van den Dool and Kratz [26]. The components of the essential oil were identified by comparing the mass spectral fragmentation patterns and the retention index values available in the Adams [27], FFNSC3 [28], NIST20 [29], and Satyal [30] databases. 

Gas chromatography with flame ionization detection (GC-FID) was carried out on *G. squarrosa* var. *squarrosa* essential oil using a Shimadzu GC 2010 equipped with a flame ionization detector (Shimadzu Scientific Instruments, Columbia, MD, USA) and using a Zebron ZB-5 GC column (60 m × 0.25 mm × 0.25 μm film thickness) (Phenomenex, Torrance, CA, USA). The same operating conditions were used for the GC-FID as those for GC-MS (above). The percent compositions were calculated from raw peak areas without standardization.

The *G. squarrosa* var. *squarrosa* essential oil was analyzed via chiral gas chromatography—mass spectrometry using a Shimadzu GCMS-QP2010S instrument (Shimadzu Scientific Instruments, Columbia, MD, USA). The mass selective detector was operated in the electron impact (EI) mode with an electron energy of 70 eV, a scan range of 40–400 atomic mass units and a scan rate of 3.0 scans per second that was fitted with a Restek B-Dex 325 chiral GC column (30 m length × 0.25 mm inner diameter × 0.25 μm film thickness) (Restek Corp., Bellefonte, PA, USA); the stationary phase was 25% 2,3-di-*O*-methyl-6-*O*-*t*-butyldimethylsilyl-β-cyclodextrin in SPB-20 poly(20% phenyl/80% dimethylsiloxane) phase with a film thickness of 0.25 μm. Helium was the carrier gas, the column head pressure was 53.6 kPa, and the flow rate was 1.00 mL/min. The injector temperature was 240 °C, the ion source temperature was 240 °C, and the interface temperature was 240 °C. The solvent cut time was 5 min. The GC oven temperature was programmed with an initial temperature of 50 °C, which was held for 5 min, then increased at a rate of 1 °C/min until a temperature of 100 °C, after which the temperature was increased at a rate of 2 °C/min to 220 °C, for a total GC acquisition time of 107 min. A 0.3-mL sample of the essential oil (5% *w*/*v* in dichloromethane) was injected using a splitting mode of 24.0:1. The compound enantiomers were determined by comparing their retention times with authentic samples obtained from Sigma-Aldrich (St. Louis, MO, USA). The ratios of enantiomers were calculated from raw peak areas.

### 3.3. Antibacterial and Antifungal Screening

The essential oil components, (+)-α-pinene, (–)-α-pinene, (–)-β-pinene, (+)-limonene, (–)-limonene, (–)-borneol, and (–)-bornyl acetate were obtained from Sigma-Aldrich (St. Louis, MO, USA) and were used as received. The compounds were screened for antibacterial activity against Gram-positive bacteria *Cutibacterium acnes* (ATCC No. 11827), *Staphylococcus aureus* (ATCC No. 29213), *Staphylococcus epidermidis* (ATCC No. 12228), *Streptococcus pneumoniae* (ATCC No. 49136), and *Streptococcus pyogenes* (ATCC No. 19615), and for antifungal activity against dermatophyte molds *Microsporum canis* (ATCC No. 11621), *Microsporum gypseum* (ATCC No. 24102), *Serratia marcescens* (ATCC No. 14756), *Trichophyton mentagrophytes* (ATCC No. 18748), and *Trichophyton rubrum* (ATCC No. 28188), using the microbroth dilution technique [31,32].

Each of the bacterial strains was cultured on tryptic soy agar medium. A 5000-μg/mL solution of each test compound was prepared in dimethylsulfoxide (DMSO, Sigma-Aldrich, St. Louis, MO, USA), and 50 μL was diluted in 50 μL of cation-adjusted Mueller Hinton broth (CAMBH) (Sigma-Aldrich, St. Louis, MO, USA), the 100-μL mixture was added to the top well of a 96-well microdilution plate. The prepared stock solution of each compound was serially two-fold-diluted in fresh CAMBH to obtain final concentrations of 2500, 1250, 625, 312.5, 156.3, 78.1, 39.1, and 19.5 μg/mL (final DMSO concentrations of 50%, 25%, 12.5%, 6.25%, 3.13%, 1.56%, 0.78%, and 0.39%). Freshly harvested bacteria with approximately 1.5 × 10^8^ colony-forming units (CFU) per mL final concentration (determined using McFarland standard) were added to each well of the 96-well microdilution plates, which were then incubated at 37 °C for 24 h. Gentamicin (Sigma-Aldrich, St. Louis, MO, USA) was used as the positive antibacterial control and DMSO was the negative control. The minimum inhibitory concentration (MIC) was determined to be the lowest-concentration well that did not show turbidity. Each assay was carried out in triplicate.

For the antifungal screening, the tested fungi were cultured on yeast malt agar (Sigma-Aldrich, St. Louis, MO, USA). Stock solutions (5000 μg/mL) of the test compounds were prepared in DMSO and diluted as above in fresh yeast-nitrogen growth medium (broth) (Sigma-Aldrich, St. Louis, MO, USA). The freshly harvested fungi, with approximately 7.5 × 10^7^ CFU/mL final concentrations in yeast-nitrogen growth medium, were added to each well of the 96-well microdilution plates and were then incubated at 35 °C for 24 h. Amphotericin B (Sigma-Aldrich, St. Louis, MO, USA) served as the positive antifungal control, while the negative control was DMSO. The antifungal assays were carried out in triplicate.

## 4. Conclusions

This is the first report on the essential oil characterization of *Grindelia squarrosa* var. *serrulata* from southern Idaho. The essential oil was rich in monoterpenoids and comparable in composition to *G. squarrosa* (variety not indicated) from western Montana, which suggests chemotype stability in North American populations; however, it was very different from *G. squarrosa* essential oils cultivated in Europe. The antimicrobial activities of the major components of *G. squarrosa* essential oil support the use of the plant to treat respiratory and dermal infections.

## Figures and Tables

**Table 1:** Chemical composition of the essential oil from the aerial parts of *Grindelia squarrosa* var. *serrulata* collected in southern Idaho.

RI_calc_	RI_db_	Compounds	%	RI_calc_	RI_db_	Compounds	%
788	780	3-Methyl-2 butenal	0.1	1392	1392	(*Z*)-Jasmone	0.1
849	849	(2*E*)-Hexenal	0.2	1429	1427	γ-Elemene	1.6
923	923	Tricyclene	0.1	1447	1447	Geranylacetone	0.1
926	925	α-Thujene	0.1	1454	1454	α-Humulene	0.2
934	933	α-Pinene	21.9	1474	1475	γ-Muurolene	0.1
950	950	Camphene	1.7	1480	1480	Germacrene D	2.4
953	953	Thuja-2,4(10)-diene	0.2	1487	1487	β-Selinene	0.3
972	971	Sabinene	0.2	1494	1497	Bicyclogermacrene	0.2
978	978	β-Pinene	4.2	1497	1500	α-Muurolene	0.1
989	989	Myrcene	1.0	1501	1504	*epi*-Zonarene	0.1
1007	1006	α-Phellandrene	0.7	1504	1503	Bornyl isovalerate	0.1
1010	1009	δ-3-Carene	0.1	1511	1510	1,11-Oxidocalamenene	0.4
1017	1017	α-Terpinene	0.3	1516	1518	δ-Cadinene	0.3
1025	1025	*p*-Cymene	0.2	1535	1540	Selina-4(15),7(11)-diene	0.2
1030	1026	Limonene	17.1	1539	1542	Selina-3,7(11)-diene	0.1
1032	1031	β-Phellandrene	0.1	1557	1557	Germacrene B	3.5
1033	1032	1,8-Cineole	0.1	1569	1571	(3*Z*)-Hexenyl benzoate	0.1
1035	1034	2,2,6-Trimethylcyclohexanone	0.1	1574	1576	Spathulenol	0.1
1035	1034	(*Z*)-β-Ocimene	tr	1577	1581	Hexyl benzoate	0.1
1046	1046	(*E*)-β-Ocimene	1.7	1583	1582	*epi*-Globulol	0.1
1057	1057	γ-Terpinene	0.1	1624	1624	Selina-6-en-4β-ol	0.1
1086	1086	Terpinolene	10.6	1626	1627	1-*epi*-Cubenol	0.1
1090	1093	*p*-Cymenene	0.1	1640	1638	τ-Cadinol	0.1
1113	1113	(*E*)-4,8-Dimethylnona-1,3,7-triene	0.2	1642	1640	τ-Muurolol	0.2
1121	1122	Chrysanthenone	0.2	1645	1644	α-Muurolol (=δ-Cadinol)	0.1
1127	1127	α-Campholenal	0.5	1653	1649	β-Eudesmol	1.4
1129	1129	1,3,8-*p*-Menthatriene	0.2	1695	1696	Juniper camphor	0.2
1141	1141	*trans*-Pinocarveol	1.0	1765	1769	Benzyl benzoate	0.2
1142	1140	*cis*-Verbenol	0.5	1990	1994	Manoyl oxide	1.3
1146	1145	*trans*-Verbenol	2.2	2226	a	Methyl grindelate	0.2
1147	1145	Camphor	0.4	2300	2300	Tricosane	0.3
1150	1150	α-Phellandren-8-ol	0.2	2356	2355	Grindelic acid	1.5
1162	1164	Pinocarvone	0.6	2400	2400	Tetracosane	0.2
1172	1170	Borneol	6.5	2500	2500	Pentacosane	1.4
1176	1176	*cis*-Pinocamphone	0.3	2600	2600	Hexacosane	0.2
1180	1180	Terpinen-4-ol	0.2	2700	2700	Heptacosane	1.0
1188	1188	*p*-Cymen-8-ol	0.2	2800	2800	Octacosane	tr
1196	1196	Myrtenal	0.7			Monoterpene hydrocarbons	60.4
1197	1194	Myrtenol	0.4			Oxygenated monoterpenoids	18.6
1208	1205	Verbenone	0.9			Sesquiterpene hydrocarbons	9.5
1217	1217	Coumaran	0.5			Oxygenated sesquiterpenoids	4.0
1220	1218	*trans*-Carveol	0.3			Diterpenoids	1.7
1285	1285	Bornyl acetate	3.3			Benzenoid aromatics	1.0
1308	1309	4-Vinylguaicol	0.2			*n*-Alkanes	3.2
1322	1322	Myrtenyl acetate	0.1			Others	1.0
1332	1334	Bicycloelemene	0.2			Total identified	99.3
1335	1336	δ-Elemene	0.3				

RI_calc_ = Retention index determined with respect to a homologous series of *n*-alkanes on a ZB-5ms column. RI_db_ = Reference retention index obtained from the databases. tr = trace (<0.05%). a—The MS fragmentation showed 85% similarity, but a reference RI was not available.

**Table 2:** Major components of *Grindelia* essential oils.

*Grindelia* Species	Geographical Origin	Major Components (>4%)	Ref.
*Grindelia discoidea* Hook. & Arn. (syn. *G. pulchella* var. *pulchella*)	Argentina	(*Z*,*E*)-Farnesol (18.2–34.9%), (*E*,*E*)-farnesol (9.0–16.8%), γ-cadinene (9.4–15.6%), globulol (6.2–10.5%), (*E*)-β-caryophyllene (trace-8.6%), δ-cadinene (3.2–6.1%)	[12]
*Grindelia hirsutula* Hook. & Arn.	Romania	Limonene (7.0%), α-pinene (6.2%), germacrene D (4.2%), spathulenol (5.5%), 10,11-epoxycalamenene (4.1%)	[17]
*Grindelia humilis* Hook. & Arn. (syn. *G. hirsutula* Hook. & Arn.)	Egypt	Polyacetylene isomer (22.1%), germacrene D (11.9%), polyacetylene isomer (10.5%), bornyl acetate (5.1%), α-pinene (4.9%), (*E*)-lachnophyllol acetate (4.1%)	[13]
*Grindelia integrifolia* DC.	Poland	Myrcene (16.9%), spathulenol (12.3%), β-eudesmol (11.9%), limonene (10.1%), α-cadinene (6.4%), α-pinene (4.6%), germacrene D (4.5%), humulene epoxide I (4.1%)	[18]
*Grindelia robusta* Nutt. (syn. *G. hirsutula* Hook. & Arn.)	Germany	Germacrene D (23.3%), α-pinene (13.4%), germacrene B (8.3%), myrcene (7.2%), (*E*)-β-caryophyllene (4.1%)	[16]
	Commercial ^a^	Borneol (14.8%), α-pinene (8.8%), *trans*-pinocarveol (6.1%), bornyl acetate (5.4%), limonene (4.1%), p-cymen-8-ol (4.1%)	[13]
	Italy	Borneol (15.2%), α-pinene (10.3%), *trans*-pinocarveol (7.0%), bornyl acetate (4.5%), limonene (4.3%), β-eudesmol (4.1%)	[14]
	Italy	Borneol (15.0%), α-pinene (11.0%), *trans*-pinocarveol (8.2%), β-eudesmol (5.5%), bornyl acetate (4.4%), β-selinene (4.3%), limonene (4.2%),	[15]
*Grindelia squarrosa* (Pursh) Dunal	Germany	Limonene (16.2%), germacrene B (13.2%), α-pinene (10.4%), phytone (6.5%), bornyl isovalerate (4.3%)	[16]
	Commercial ^a^	Limonene (16.8%), α-pinene (16.1%), germacrene D (6.8%), β-pinene (5.2%), borneol (4.5%)	[13]
	Romania	Bornyl acetate (10.8%), α-pinene (8.3%), limonene (8.1%), spathulenol (5.4%), caryophyllene oxide (4.9%)	[17]
	Montana, USA	α-Pinene (23.2%), borneol (16.6%), limonene (14.7%), *p*-cymen-8-ol (5.8%), bornyl acetate (5.1%)	[19]
	Idaho, USA	α-Pinene (21.9%), limonene (17.1%), terpinolene (10.6%), borneol (6.5%), β-pinene (4.2%)	this work

^a^ Commercial sample, purchased in Germany, but geographical source not indicated.

**Table 3:** Enantiomeric distribution of terpenoids in *Grindelia squarrosa* var. *serrulata* essential oil.

Compounds	RT_std_	RT_EO_	%
(–)-α-Pinene	15.92	15.19	99.3
(+)-α-Pinene	16.40	16.38	0.7
(–)-Camphene	17.73	17.86	99.0
(+)-Camphene	18.30	18.40	1.0
(+)-Sabinene	19.74	19.78	100.0
(–)-Sabinene	20.60	nd	0.0
(+)-β-Pinene	20.27	20.28	10.7
(–)-β-Pinene	20.62	20.62	89.3
(–)-α-Phellandrene	22.59	nd	0.0
(+)-α-Phellandrene	22.81	22.85	100.0
(–)-Limonene	25.06	25.09	2.8
(+)-Limonene	25.99	25.39	97.2
(–)-β-Phellandrene	26.15	26.50	29.4
(+)-β-Phellandrene	26.88	27.06	70.6
(–)-Camphor	49.84	50.12	100.0
(+)-Camphor	50.34	nd	0.0
(–)-Borneol	58.59	58.31	100.0
(+)-Borneol	59.11	nd	0.0
(–)-Bornyl acetate	59.46	59.48	100.0
(+)-Bornyl acetate	na	nd	0.0
(–)-α-Terpineol	59.73	59.79	80.2
(+)-α-Terpineol	60.58	60.62	19.8
(–)-Verbenone	61.70	61.84	100.0
(+)-Verbenone	na	nd	0.0
(+)-Germacrene D	73.48	73.52	78.5
(–)-Germacrene D	73.73	73.78	21.5

RT_std_ = Retention time for standard compounds in minutes, RT_EO_ = Retention time for the essential oil in minutes, na = standard compound not available, nd = compound not detected.

**Table 4:** Antibacterial and antifungal activities (MIC, μg/mL) of essential oil components.

	Bacteria				
Compound	*Cutibacterium* *acnes*	*Staphylococcus aureus*	*Staphylococcus epidermidis*	*Streptococcus pneumoniae*	*Streptococcus* *pyogenes*
(+)-α-Pinene	625	625	312.5	78.1	625
(–)-α-Pinene ^a^	625	312.5	312.5	78.1	312.5
(–)-β-Pinene ^a^	312.5	156.3	312.5	39.1	625
(+)-Limonene ^a^	625	312.5	312.5	78.1	312.5
(–)-Limonene	39.1	312.5	78.1	78.1	625
(–)-Borneol ^a^	312.5	78.1	312.5	625	625
(–)-Bornyl acetate ^a^	312.5	312.5	312.5	312.5	625
Gentamicin ^b^	<19.5	0.61	<19.5	<19.5	<19.5
DMSO ^c^	1250	1250	1250	1250	1250
	**Fungi**				
	***Microsporum*** ***canis***	***Microsporum gypseum***	***Serratia*** ***marcescens***	***Trichophyton mentagrophytes***	***Trichophyton rubrum***
(+)-α-Pinene	312.5	156.3	312.5	156.3	312.5
(–)-α-Pinene ^a^	312.5	312.5	312.5	312.5	312.5
(–)-β-Pinene ^a^	312.5	312.5	312.5	156.3	312.5
(+)-Limonene ^a^	312.5	312.5	625	312.5	312.5
(–)-Limonene	312.5	156.3	312.5	156.3	312.5
(–)-Borneol ^a^	312.5	312.5	625	156.3	312.5
(–)-Bornyl acetate ^a^	312.5	312.5	625	156.3	312.5
Amphotericin B ^b^	<19.5	<19.5	<19.5	<19.5	<19.5
DMSO ^c^	1250	1250	1250	1250	1250

^a^ Major enantiomer. ^b^ Positive control. ^c^ Dimethylsulfoxide, negative control.

## Data Availability

All data are available in the article.
